# Time-resolved multidimensional NMR with non-uniform sampling

**DOI:** 10.1007/s10858-013-9811-1

**Published:** 2014-01-17

**Authors:** Maxim Mayzel, Joakim Rosenlöw, Linnéa Isaksson, Vladislav Y. Orekhov

**Affiliations:** The Swedish NMR Centre, University of Gothenburg, Box 465, 40530 Göteborg, Sweden

**Keywords:** IDP, Post-translational modification, NUS, PARAFAC, MDD, ITAM, CD79, BEST-TROSY, Real-time

## Abstract

Time-resolved experiments demand high resolution both in spectral dimensions and in time of the studied kinetic process. The latter requirement traditionally prohibits applications of the multidimensional experiments, which, although capable of providing invaluable information about structure and dynamics and almost unlimited spectral resolution, require too lengthy data collection. Our work shows that the problem has a solution in using modern methods of NMR data collection and signal processing. A continuous fast pulsing three-dimensional experiment is acquired using non-uniform sampling during full time of the studied reaction. High sensitivity and time-resolution of a few minutes is achieved by simultaneous processing of the full data set with the multi-dimensional decomposition. The method is verified and illustrated in realistic simulations and by measuring deuterium exchange rates of amide protons in ubiquitin. We applied the method for characterizing kinetics of in vitro phosphorylation of two tyrosine residues in an intrinsically disordered cytosolic domain of the B cell receptor protein CD79b. Signals of many residues including tyrosines in both phosphorylated and unmodified forms of CD79b are found in a heavily crowded region of 2D ^1^H–^15^N correlation spectrum and the significantly enhanced spectral resolution provided by the 3D time-resolved approach was essential for the quantitative site-specific analysis.

**Electronic supplementary material:**

The online version of this article (doi:10.1007/s10858-013-9811-1) contains supplementary material, which is available to authorized users.

## Introduction

NMR spectroscopy is a versatile tool for studying macromolecular dynamics. Remarkable progress has been demonstrated in studies of equilibrium conformatinal transitions (Baldwin and Kay 2009), protein folding (Frieden et al. 1993; Balbach et al. 1995; Rennella et al. 2012b), post-translational modifications (Selenko et al. 2008; Liokatis et al. 2010; Theillet et al. 2012a) and other kinetic processes (Smith et al. 2013). The time-resolved NMR (TR NMR) spectroscopy allows monitoring and quantification of a kinetic process occurring at the time scale of hundreds of milliseconds and longer. TR NMR was used for characterizing amide deuterium exchange (Dempsey 2001; Rennella et al. 2012a), protein and RNA folding (Balbach et al. 1996; Dyson and Wright 2004; Zeeb 2004; Furtig et al. 2007; van Nuland et al. 2008; Roche et al. 2013), enzymatic activity (Smith et al. 2013), post-translational modifications (Landrieu et al. 2006; Selenko et al. 2008; Liokatis et al. 2010, 2012; Theillet et al. 2012a; Cordier et al. 2012) and in other cases. Early applications of TR NMR to protein folding (Frieden et al. 1993; Balbach et al. 1995) were performed using one-dimensional (1D) spectra, which are the most sensitive and fastest experiments. In many practical cases the duration of a 1D experiment is an order of magnitude shorter than the time of the studied reaction and the kinetics parameters can be obtained from fitting the peak intensities to an appropriate model. It is clear, however, that signal resolution in the 1D spectrum is not sufficient for large molecules and applications of the two-dimensional (2D) spectroscopy in kinetic studies were presented (Balbach et al. 1996). Increasing dimensionality of the experiment dramatically alleviates the signal overlap, but lead to lengthy measurements, which may surpass time of the reaction and, thus, complicate the analysis. With traditional uniform sampling of the signal in the time domain, the number of collected data points and the corresponding duration of the experiment increase exponentially with the number of spectrum dimensions and polynomially with the digital resolution. Moreover, extra resolution in the indirect spectral dimensions comes at the price of sensitivity, since for uniform sampling the measurement time is equally allocated for the short and long acquisition times, where the signal-to-noise ratio may differ significantly.

A first solution to the problem was suggested by Balbach et al. (1996; Landrieu et al. 2006). Instead of collecting a series of short spectra during the course of the reaction, a single relatively long 2D experiment was run concurrently with the reaction. In this setup, the signal evolution in the indirect spectral dimension is modulated both by the signal chemical shift and the reaction dependent peak intensity. During the reaction, intensities of the NMR signals corresponding to the reaction substrates decrease, which leads to additional broadening of the corresponding peaks. The peaks corresponding to reaction products, for which the concentration increases during the reaction, have complex shapes with positive intensity in the middle and negative flaps on the both sides. The reaction kinetic parameters are obtained from the line shape analysis of the spectra. A similar approach was recently extended to the 3D case and demonstrated by the characterization of a long-lived folding intermediate of amyloidogenic protein β2-microglobulin (B2 M) (Rennella et al. 2012b). A fast pulsing 3D BEST-TROSY HNCO (BT-HNCO) experiment was run concurrently with the transition from the intermediate to the native protein state. Duration of the experiment, 40 min, was longer than the half-life of the reaction intermediate, about 20 min, and the reaction kinetic parameters were encoded in the peak line shapes in one of the indirect dimensions.

The approach described in the previous paragraph has its caveats, since the distorted line shapes decrease resolution and sensitivity in the spectra. Besides, the line shape analysis may be difficult for non-first order reaction with complex kinetics (Theillet et al. 2013). An alternative solution is to decrease measurement time of the individual experiment in order to make it shorter than the reaction time. This brings TR NMR to the realm of the fast spectroscopy, which finds optimal balance between sensitivity, resolution and experiment time (Hoch and Stern 2001; Kupce et al. 2003; Zhang and Brüschweiler 2004; Hiller et al. 2005; Billeter and Orekhov 2012; Kazimierczuk et al. 2013; Lee et al. 2013). In cases when the sensitivity is in abundance, the single scan 2D methods allow studying processes with times as short as a fraction of a second (Gal and Frydman 2006; Gal et al. 2007, 2009). In many practical applications, however, the reaction time is or can be tuned to the range from a few minutes to tens of hours. Typical examples are the amide proton hydrogen–deuterium exchange in globular proteins and chemical reactions of post-translational modifications in proteins. In this time scale, it is common to consecutively record series of two-dimensional ^1^H–^15^N correlation spectra, which, especially in the SOFAST-HMQC version (Schanda and Brutscher 2005) (Amata et al. 2013) are optimized for high sensitivity and short measurement time.

As complexity of the studied biological systems increases, resolution provided by the 2D SOFAST-HMQC experiments becomes insufficient for resolving critical resonances in the spectra. The situation is exemplified in this study by the phosphorylation of tyrosine residues in the intrinsically disordered cytosolic domain of CD79b, a signalling component of the B-cell receptor. Upon activation of the receptor, the two tyrosines located in the immunoreceptor tyrosine-based activation motif (ITAM) of CD79b are phosphorylated by Src-family tyrosine kinases (Johnson et al. 1995; Monroe 2006). Phosphorylation of the ITAM tyrosines in B- and T-cell receptors is one of the earliest events in B- and T-cell signalling and disruption or deregulation of the phosphorylation is implicated in multitude of diseases and pathogenic states. Signals in the 2D ^1^H–^15^N spectrum of CD79b are heavily overlapped, which is typical for intrinsically disordered proteins. Unlike serine and threonine residues, for which amide signals acquire distinct chemical shifts upon phosphorylation (Bienkiewicz and Lumb 1999), tyrosine peaks move only moderately in the 2D ^1^H–^15^N correlation spectra from their non-phosphorylated positions (Theillet et al. 2012b). For CD79b, signals of both tyrosine forms are found in crowded spectral region. Moreover, high signal overlap in the 2D spectra complicates quantification of kinetic effects sensed by other residues, which could report on the reaction intermediates and reveal possible coupling between the phosphorylation sites. Thus, it is desirable to use 3D or even higher dimensional experiments, but the measurement time must be comparable to the currently used fast 2D spectra. This corresponds to the time saving of one-two orders of magnitude relative to a traditional 3D experiment.

Recent, advances in non-uniform sampling (NUS) and novel signal processing methods allow acquisition of three and higher dimensional spectra in very short time. For NUS, the number of measured data points is related to the number of signals in the spectrum and is largely independent of the spectra resolution and dimensionality (Jaravine et al. 2006). In comparison to uniform sampling, only a small fraction of the data points is acquired with NUS. Nowadays, the NUS method is routinely used in studies of protein structure and dynamics (Luan et al. 2005a; Tugarinov et al. 2005; Hiller et al. 2009; Lemak et al. 2010; Takeuchi et al. 2010; Sun et al. 2012; Hyberts et al. 2012; Mobli et al. 2012; Coggins et al. 2012; Stanek et al. 2013; Isaksson et al. 2013; Kazimierczuk et al. 2013). In most of the NUS applications, the peak intensities (or integrals) are used in qualitative or semi-quantitative way. It has been demonstrated that the high resolution achieved with NUS improves accuracy in determining peak positions (Jaravine et al. 2006), which is particularly helpful, for example when measuring scalar and residual dipolar couplings (Thiele and Bermel 2012). However, quantitative analysis of kinetics in the TR NMR experiments requires accurate signal intensities, which should precisely follow the changes in concentration of the substrates and products of the reaction. Linearity of the peak intensities in the NUS spectra reconstructions were reported for most of the NUS processing methods, including Forward Maximum Entropy, SSA (Stanek et al. 2013), SCRUB (Coggins et al. 2012), SIFT (Matsuki et al. 2011), multi-dimensional decomposition (MDD) (Luan et al. 2005a), and Compressed Sensing (Kazimierczuk and Orekhov 2011, 2012; Holland et al. 2011). However, NUS only finds its way in quantitative applications and more practical demonstrations are needed to find optimal sampling and processing regimes for different time saving methods.

In this work we demonstrate that the combination of fast pulsing sensitivity optimized 3D BEST-TROSY type experiments with NUS and co-processing using multidimensional decomposition allows quantitative time-resolved kinetic studies with the reaction time resolution down to a few minutes. The method is validated by realistic simulations and measurement of amide proton deuterium exchange in ubiquitin and exemplified by determining kinetics of in vitro phosphorylation of the two ITAM tyrosines of CD79b.

## Theory

### Accumulation of the time-resolved NUS experiment

In the traditional uniform sampling scheme, measurements are performed at regular time points separated by a dwell-time delay, which value is given by the inverse of the spectral width. The total number of measured data points for the uniform sampling is defined by the digital resolution of the spectrum. Thus, the spectral width and digital resolution unambiguously define the time sampling grid, which is often referred as the Nyquist grid. In NUS, the measurements are performed only for a (small) fraction of randomly selected points from the Nyquist grid. In order to increase sensitivity of the experiment, more points are selected where the signal has the highest amplitude. In this study, all the sampling schedules were generated using the program *nussampler*, which is part of the *mddnmr* software (Orekhov and Jaravine 2011).

**Fig. 1:**
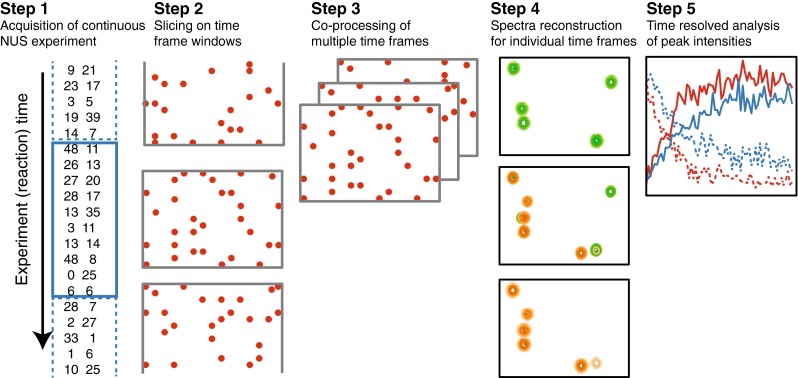
Schematic overview of the time-resolved analysis using non-uniform sampling and co-MDD processing (see text for details)

In the traditional time resolved experiment, series of spectra are recorded concurrently with the reaction progress and the decision has to be made prior to the experiment about the duration of the individual spectra in the set. This may be a difficult task when the reaction rate is unknown or when several processes with different rates occur simultaneously. The later case is typical, for example, when measuring the deuterium-hydrogen exchange and for studies of protein post-translational modifications with multiple modification sites. In this work we suggest a new approach illustrated in Fig. 1. An uninterrupted experiment is acquired with a randomized NUS schedule over the entire reaction period. The time windows corresponding to the individual spectra are sliced from the full experiment only at the stage of the data processing and their durations can be optimized for the specific reaction rates. We compared two modes of the time-window selection. The first one is linear, with constant window sizes (WS). The second mode is exponential, where size of the first window is small and every next window contains more measured points than the previous one by a constant factor (ΔWS). In both cases, the set of spectra corresponding to the sliced windows is processed using the co-MDD method described below. The constant window method is the simplest and corresponds to the traditional time-resolved setup, where a set of experiments with identical parameters is acquired during the reaction. The exponential window approach reduces the number of windows and adjustable parameters in the co-MDD, while preserving good time-resolution for the critical initial period of the reaction.

We should emphasize the difference between the NUS method described above and the approach suggested by Balbach and co-workers (Balbach et al. 1996). In both cases, one long experiment is accumulated over the whole reaction period. The later approach, results in one spectrum, where information about the reaction kinetics is coded in the distortions of the peak line-shapes. In the NUS method, however, the time course of the reaction is obtained from the analysis of the peak intensities (integrals) in the set of traditionally looking spectra corresponding to individual time points of the reaction.

### Co-MDD analysis of the time-resolved spectra

For most multidimensional NMR spectra it is reasonable to assume that a peak in the spectrum is completely described by its one-dimensional line-shapes in all spectral dimensions. For example, the 3D HNCO experiment can be approximated by the following model:

1 $$ S^{k}_{HNCO} = \sum_{i} {a_{i} F_{i}^{H}\,{\otimes}\,F_{i}^{CO}\,\otimes\,F_{i}^{N} } $$

where the model spectrum ***S***_*HNCO*_ is given by the sum of fixed number of components *N*_*c*_ enumerated by index *i* = *1…N*_*c*_. Each component *i*, which in this case represents a peak in the 3D HNCO, is given by the product of the amplitude *a*_*i*_ and three normalized one-dimensional shapes *F*_*i*_^*H*^, *F*_*i*_^*CO*^, and *F*_*i*_^*N*^ for the amide proton, carbonyl carbon, and amide nitrogen spectral dimensions, respectively. The symbol ⊗ denotes the outer product operation, which in Eq. 1 produces a 3D matrix from three one-dimensional shapes. Equation 1 defines the model of the multi-dimensional decomposition (MDD) (Orekhov et al. 2001). Notably, Eq. 1 is valid for frequency domain, time domain, and combination of both. The model can be generalized for a set of several experiments (Korzhnev et al. 2001; Damberg et al. 2002; Gutmanas et al. 2004; Luan et al. 2005b), e.g. for this study, those consecutively recorded for the same sample in the time resolved experiment. The assumption is that the signals in all the experiments in the set have the same positions and line-shapes and may differ only in their amplitude. This assumption is fulfilled when temperature, pH, and other general protein solution conditions do not significantly change in the course of the studied reaction. Let us consider several 3D HNCO spectra enumerated by the index *k* = *1…K*:

2 $$ S^{k}_{HNCO} = \sum_{i} {a_{i}^{k} F_{i}^{H} \otimes F_{i}^{CO}
\otimes F_{i}^{N} } $$

All 3D spectra in Eq. 2 share shapes for all three dimensions and can be combined into one 4D data set

3 $$ S_{4DHNCO} = \mathop \sum \limits_{i}  A_{i} \otimes F_{i}^{H} \otimes F_{i}^{CO} \otimes F_{i}^{N} $$

where vector *A*_*i*_ = (*a*_*i*_^1^, *a*_*i*_^2^,…,*a*_*i*_^*K*^). Equation 3 defines co-MDD model for *K* spectra recorded in the time-resolved experiment. The 4D MDD model parameters, i.e. amplitudes *A*_*i*_ and time domain shapes *F*_*i*_^*H*^, F_i_^*CO*^, *F*_*i*_^*N*^ in Eq. 3 are found using general *N*-dimensional decomposition algorithm implemented in the *mddnmr* software (Orekhov and Jaravine 2011). Then, the individual 3D experiments, which correspond to different time windows during the reaction, are reconstructed using Eq. 2.

Using co-processing of series of NMR experiments, good N-D spectra can be obtained with only few data points, often <1 % of the measurements required for the conventionally sampled 3D experiment (Jaravine et al. 2008). When strong signal is present in several spectra of the set, the common shapes *F*_*i*_^*H*^, *F*_i_^CO^, *F*_*i*_^*N*^ are well defined and only the amplitudes of the peaks—*a*_*i*_ need to be determined separately for individual 3D HNCO spectra. However, for rapidly decaying signals, the peaks are observed only in a few first spectra. This may be insufficient for defining the common shapes. To overcome this limitation in the current work we used two different approaches. In the case of hydrogen–deuterium exchange experiment on ubiquitin, the sample solution contained 20 % of H_2_O. Thus, even for the rapidly exchanged amides, peaks did not vanish completely in the course of the reaction but levelled off at approximately 20 % of the maximum intensity. For the CD79b phosphorylation studies we had acquired a short reference HNCO spectrum prior to the reaction start and used this spectrum as a zero time point in the co-processing. This spectrum serves to define the shapes for the peaks corresponding to the non-phosphorylated CD79b. The shapes for the phosphorylated form are well defined by the measurements at the end of the reaction.

### Determination of the phosphorylation rates

Following the co-MDD calculation, reconstructed 3D spectra for individual reaction time windows are produced and the peak intensities for each signal are estimated using the *seriesTab* script included in the *nmrPipe* software (Delaglio et al. 1995). The reaction kinetic parameters are obtained using weighted non-linear least-square fitting with exponential function.

3 $$ A(\tau ) = A_{0} *exp^{ - k*\tau } + A_{l} $$

where *A*_*0*_ is the initial signal intensity, *k* is the apparent first order reaction rate, and *A*_*l*_ is the peak intensity plateau value. For CD79b residues that exhibit different signals in the phosphorylated and unmodified states, simultaneous fit of the two signals was performed for the model:

4 $$ \left[ {A^{u} \left( \tau \right); A^{p} \left( \tau \right)} \right] = \left[ {A_{o}^{u} *exp^{ - k*\tau } + A_{l}^{u} ;A_{o}^{p} *exp^{k*\tau } + A_{l}^{p} } \right] $$

where *k* is the apparent first order reaction rate (Theillet et al. 2013) and *p* and *u* superscripts denote phosphorylated and non-phosphorylated (unmodified) states signals, respectively. The weighting factors in the non-linear least-square fit are set proportionally to the square root of the time window size.

### Simulation of the kinetic NUS experiment

The simulations with well-defined signal properties and experimental spectral noise are used for estimating accuracy and precision of the obtained reaction kinetic parameters. Comparisons of the accuracies obtained for different signal-to-noise ratio (SNR) are used to assess robustness of the proposed methodology against noise. We also checked if the parameter errors obtained in the weighted non-linear fitting are good proxies for the actual accuracy and precision of the derived reaction rates. Finally, we examine the effect of co-processing with a reference spectrum containing strong signals with stationary intensities.

Success of the presented time-resolved analysis depends on the rate of the studied reaction, the total duration of the experiment, and SNR. The latter parameter changes in time with signal intensity following concentration of the reaction substrates and products. In order to quantify the SNR in this study, we define steady-state signal-to-noise ratio (ss-SNR) as the SNR value for a spectrum recorded over the period of the complete TR-experiment assuming zero rate for the reaction, i.e. for constant signal intensity.

The simulations are performed for three different ss-SNR values (340, 170, and 85), two different initial window sizes (16 and 64 hyper complex points), and four window size increments (20, 10, 5 and 0 %). In each calculation, 14 non-overlapping signals representing different deuterium exchange with rates ranging from 3.0 × 10^−4^ to 2.8 × 10^−1^ min^−1^ are generated for 16 NUS schedules. The signals have equal amplitudes at the time point zero and decay to a plateau value of 20 % of the original intensity. The largest of the ss-SNR values used in the simulations (340) corresponds to the average steady state peak amplitude of slowly decaying signals in the ubiquitin deuterium exchange (HD) experiment. The simulated signals were combined with noise extracted from an empty region of the HD experiment. For each ss-SNR, 16 synthetic data sets were created using different random seeds for generating NUS schedules. Statistics over analysis of the 16 synthetic time-resolved experiments allowed us to calculate for every signal mean fit error, precision (as standard deviation of decay rates) and accuracy (as root mean square deviation of the decay rates from the correct value). Simulations of co-processing with the reference spectrum were performed for the ss-SNR of 85.

## Materials and methods

### In-vitro production and phosphorylation of ^15^N/^13^C labeled cytoplasmic domain of human CD79b

^15^N/^13^C labeled cytoplasmic domain of human CD79b (CD79b_cyt_) was produced using an in-house developed cell-free expression system as previously described (Isaksson et al. 2013). This construct includes residues 181–229 from the full CD79b and two additional residues Ser and Leu at the N-terminus. Phosphorylation was done in vitro using recombinant Src-family tyrosine kinase Fyn (Life technologies). Purified and lyophilized CD79b_cyt_ was dissolved to a final concentration of 90 μM in aqueous buffer containing 20 mM sodium phosphate, 150 mM sodium chloride, 1× Complete EDTA-free protease inhibitor cocktail (Roche), 15 % D_2_O, 10 mM MgCl_2_ and 1 mM ATP at pH 7.0. The sample (150 μl) was transferred to a 3 mm Shigemi tube. Fyn kinase (and an additional 1 mM of ATP) was injected directly to the tube to a final concentration of 60.6 nM resulting in the activity of 600 units as calculated using the certificate of analysis from Life technologies. Directly after addition of the kinase the sample was placed in the spectrometer magnet at 25 °C and a 3D BT-HNCO experiment (described below) was recorded over 22 h as the phosphorylation reaction progressed. Successful phosphorylation was verified through observations of chemical shift changes displayed by the two tyrosines and neighbouring residues.

### H/D exchange studies on ubiquitin

^15^N/^13^C labeled ubiquitin (VLI research Inc) was lyophilized from 250 μl aqueous buffer containing 20 mM sodium phosphate, pH 6.0, 0.02 % sodium azide, 2 mM EDTA, 10 % D_2_O. The powder was initially dissolved in 45 μl of H_2_O and transferred into 5 mm Shigemi tube. Following injection of 180 μl of D_2_O, the sample with final ubiquitin concentration of 400 μM was rapidly placed inside the NMR magnet at 20 °C and a 3D BT-HNCO experiment was started. Taking into account the sample handling, temperature stabilization, gradient shimming, and the pulse sequence compilation the dead time between injection and data acquisition was around 5 min.

### NMR measurements

BEST-TROSY type 3D HNCO experiments (Favier and Brutscher 2011) NMR were performed on an Agilent spectrometer with Larmor frequency of 600 MHz equipped with a cryogenically cooled pulse-field gradient triple-resonance probe. For improving sensitivity, the spectra were acquired using NUS with the sampling probability density tailored to the assumed ^13^CO transverse relaxation time of 20 ms. The NUS schedules were prepared by the program *nussampler* from *mddnmr* software package (Jaravine et al. 2008; Orekhov and Jaravine 2011).

For CD79b phosphorylation: the spectral width and acquisition times for the ^13^C and ^15^N spectral dimensions were 2,262 Hz, 22 ms and 1,520 Hz, 28 ms, respectively. The sampling schedule of the 22 h experiment contained 13,945 hyper complex points, which is 6.6 times larger than the number of points in the experiment Nyquist grid. Prior to the phosphorylation, a reference spectrum with the same parameters and shorter sampling schedule was recorded during 6.7 h. The sampling schedule for the reference spectrum contained 4,214 hyper complex points. For measuring deuterium exchange on ubiquitin: the spectral width and acquisition times for the ^13^C and ^15^N spectral dimensions were 1,508 Hz, 42 ms and 1,822 Hz, 35 ms, respectively. The sampling schedule contained 12,288 hyper complex points (3.0 times oversampling). The total experiment time was 20.5 h with 5.9 s per hyper-complex data point.

## Results and discussion

### Simulations

**Fig. 2:**
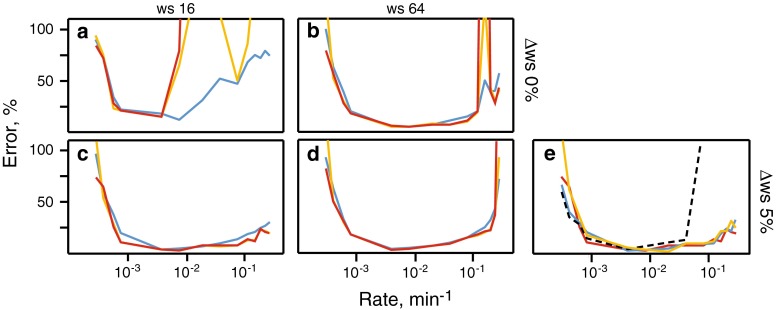
Fidelity of the time-resolved co-MDD processing for the analysis of non-uniformly sampled NMR spectra: **a**–**d** dependence of the relative fitting error (*blue*), accuracy (*red*) and precision (*orange*) are shown versus signal decay rates for steady state signal-to-noise ss-SNR = 170; **a**, **b** constant time-window sizes of 16 and 64 NUS data points, respectively, **c**, **d** exponentially increasing (5 %) time-window with initial window sizes of 16 and 64 NUS data points, respectively. **e** Reaction rate accuracies obtained using processing setup as used for **c** for ss-SNR values of 350 (*orange*), 170 (*red*), and 85 (*dashed black*); for (*blue)* curve, the data set with ss-SNR = 85 was co-processed with a reference spectrum corresponding to the zero time point of the reaction. For easy comparison with other *panels*, all accuracies were scaled to ss-SNR = 170 assuming the linear dependence of the accuracy on the signal-to-noise ratio

Intuitively it is clear that the duration of a time resolved experiment should exceed the time of the studied process, while pace of the data collection should match its rate. Since the rate decreases towards the end of the reaction, it may be beneficial to correspondingly slow down the data collection. Figure 2 shows results of the simulations, which confirm these thoughts. The figure shows errors in the fitted reaction rates as a function of the reaction rate value, initial time-window size (WS), and its increment (ΔWS). Figure 2a illustrates that usage of too small constant WS (16 hyper-complex points, 1.56 min) gives very poor results, i.e. unacceptably high fitting errors and low precision and accuracy. Larger WS (64 hyper-complex points, 6 min) (Fig. 2b) rescues the analysis, but leaves relatively high errors for the fast rates. The results improve significantly when we introduce a small window increment factor (ΔWS = 5 %). This allows small initial WS and may explain the lower errors for fast reaction rates shown in Fig. 2c. Similar results are obtained for ΔWS = 10 and 20 %, thus, the exact value of the window increment is not important. However, as expected, comparison of Fig. 2c, d shows that increase in WS leads to higher errors for the fast rates. For the slow reaction rates, the errors rise rapidly when the inverse of the reaction rate becomes comparable to the total experiment time (20.5 h).

Another question, which we address with the simulations, is if the errors obtained in the exponential least-square fit using the covariance matrix approach and those calculated as the precision over the resampling trials can be considered as a reliable estimates of the accuracy for the obtained reaction rates. In Fig. 2a, b, c, d, the mean fitting error, precision and accuracy are depicted as different lines. It is clear that both the fitting error and precision are close to the accuracy for slow and medium reaction rates as long as the errors are small. For the faster rates and overall larger errors, precision gives more realistic estimates of the accuracy, which may justify use of the resampling-based analysis for the error estimation (Isaksson et al. 2013). Similarity of the accuracy and precision indicates that the bias (if any) in the reaction rates is smaller than the experimental errors. Specific comparison of the calculated and correct rates (not shown) did not reveal any bias.

Figure 2e shows results of the simulations for different signal-to-noise values. It is clear that for the lowest ss-SNR of 85, co-MDD processing gives poor results for the fast decaying signals (*black dashed line*). The result is significantly improved (*red line*), when this data is co-processed with the reference spectrum. This is fully in line with the theory of MDD processing. The reference spectrum helps the MDD algorithm to correctly define the line shapes of the peaks, which are the same in all co-processed spectra. As the shapes are defined, the task of determining of the peak amplitudes in the individual spectra is much more easy.

Conclusively, the realistic simulations show that the proposed time-resolved co-MDD processing of high-resolution 3D HNCO spectra allows accurate and robust measurements of the reaction rates up to few inverse minutes.

### Amide H–D exchange

**Fig. 3:**
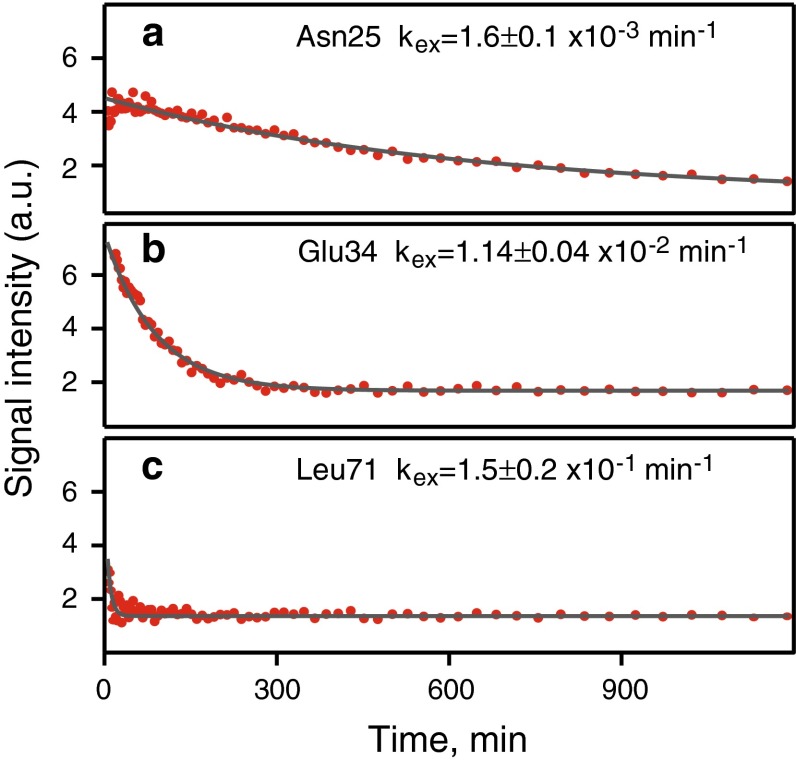
Amide hydrogen–deuterium exchange plots for three representative ubiquitin residues exhibiting **a** slow, **b** intermediate and **c** fast exchange rates. Experimental points reflect peak intensity measured in the individual time windows sliced from the 3D BT-HNCO spectrum and processed with co-MDD. Size of the first window *WS*_*0*_ was 16 points and each next window increased by 5 %, i.e. *WS*_*i*+*1*_ = 1.05 *WS*_*i*_. Lines and exchange rates correspond to best fit to Eq. 3. The residue numbers and rates of the calculated HD-exchange are annotated in the *panels*

Rates of the amide hydrogen–deuterium exchange are important indicators of protein structure, stability, and interactions (Dempsey 2001). Values of the exchange rates strongly depend on local protein structure, temperature and pH and span time scale from fraction of a second to months. While quantification of the slow exchange with characteristic times of several hours and more is not problematic from the spectroscopy point of view and are usually obtained from the traditional time-resolved experiments, measuring of the rates in the range of seconds and minutes is challenging and requires fast spectroscopy approaches (Bougault et al. 2004; Gal and Frydman 2006; Gal et al. 2009; Rennella et al. 2012b). In this work we demonstrate that 3D NUS BT-HNCO experiment with co-MDD processing can be used for quantification of the HD-exchange rates with half-times from a couple of minutes and larger. A single continuous 3D BT-HNCO experiment with randomized NUS schedule was started immediately after initiation of the hydrogen to deuterium exchange reaction by diluting concentrated aqueous protein solution by the excess (4:1) of D_2_O. The resulting data set was processed with co-MDD using constant and incremented time-window methods. Figure 3 illustrates the non-linear exponential fit for three representative residues Asn25, Glu34, and Leu71. Supplementary figure S1 and table S1 summarize the exchange rates measured in the range from 4.9 × 10^−4^ min^−1^ for Ile13 to 4.1 × 10^−1^ min^−1^ for Asn60. Total 34 amides were quantified with the fitting errors smaller than 50 %. For other amides, the exchange was either to fast or too slow at the used experimental conditions. Values presented in Table S1 generally agree well with the previously published data (Pan and Briggs 1992; Bougault et al. 2004). The HD exchange rates and fitting errors obtained using incremented and constant time-window (Table S1) are very similar. The only exception is Asn60, which has the fastest of the measured exchange rates. For this residue, the acceptable fitting error was obtained only using the incremental window. For fast exchanging residues Glu16, Ser20, Thr66 and Leu71 fits were successful in both approaches but the fitting errors were significantly lower for the incremental time-window. The results obtained with the two types of time windows are in line with the simulations, which show that the constant and incremental windows give similar results for the slow and medium rates but the incremental windows are better for quantification of fast processes.

### Phosphorylation of ITAM tyrosines in CD79b

**Fig. 4:**
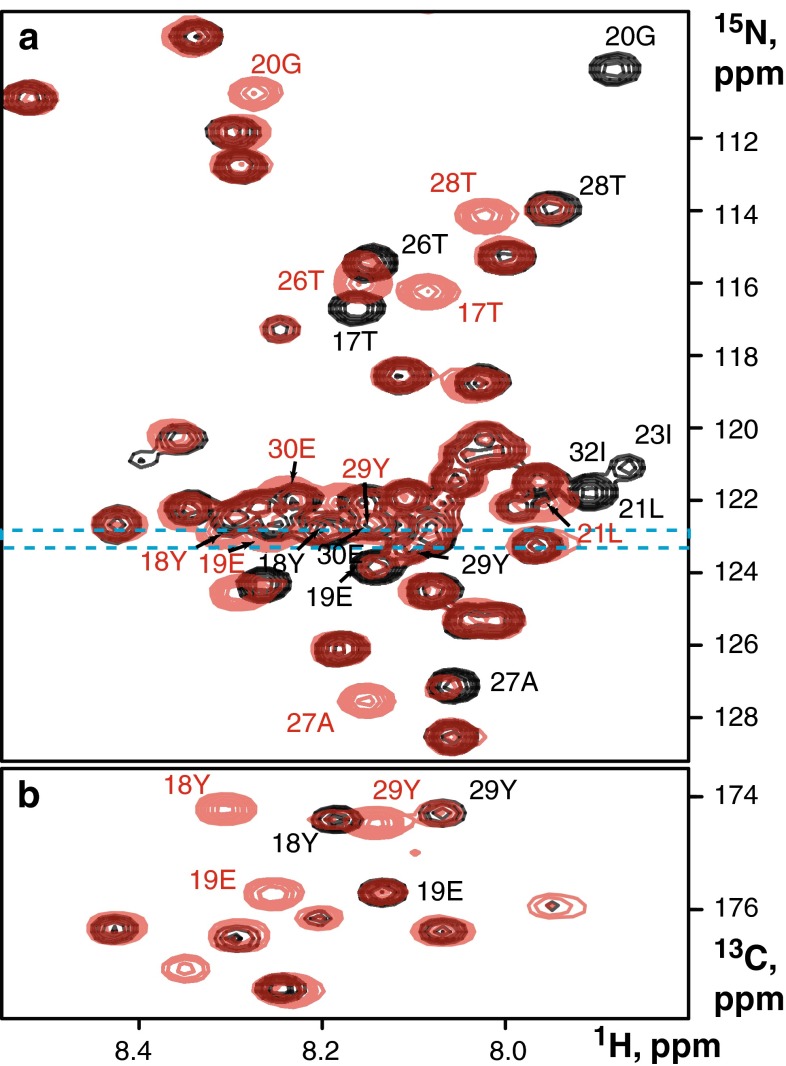
Spectra of intrinsically disordered CD79b cytosolic domain in unmodified (*black*) and phosphorylated (*red*) forms. **a** Overlay of 2D ^1^H–^15^N correlation spectra. The dashed lines indicate one-ppm-wide band centred at 123 ppm in ^15^N spectral dimension. **b** 2D ^1^H–^15^CO projections of 3D BT-HNCO spectra taken over 1 ppm-wide slice in ^15^N dimension as indicated in *panel* (**a**). Signals exhibiting significant chemical shift changes upon phosphorylation are annotated using the corresponding colours

**Fig. 5:**
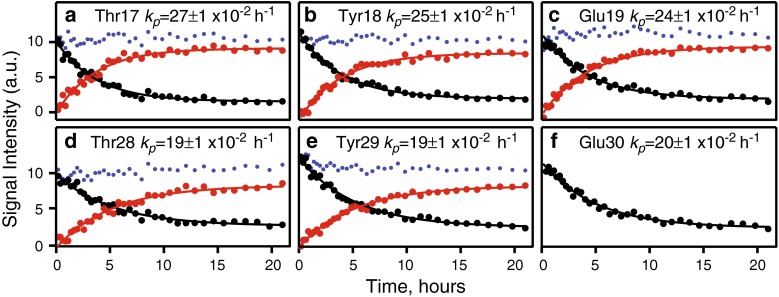
Phosphorylation of CD79b. Signal intensities in the 3D BT-HNCO spectrum for **(b)** Tyr18 and **(e)** Tyr29 and **(a**, **c**, **d**, **f)** directly adjacent residues are plotted versus time of the reaction for the unmodified (*black*) and phosphorylated (*red*) forms of the protein. The *(blue)* points designate sum of the two signal intensities. Apparent first order rates *k*_*p*_ of the reactions are annotated in the *panels*. *Lines* and exchange rates correspond to best fit to Eq. 4. For residue Glu30, phosphorylated signal strongly overlaps with Asp15, which prevents accurate intensity quantification

Understanding the function of intrinsically disordered proteins in signalling is contingent upon knowing timing and sequence of the chemical modification events. Upon ligand binding to the extracellular part of the B-cell receptor, the immunoreceptor tyrosine-based activation motifs (ITAM) in the cytosolic domains of CD79a and CD79b are phosphorylated, an event leading to initiation of the downstream intracellular signalling cascade. NMR is the most powerful method for characterising site-specific status and time sequence of the phosphorylation in proteins with multiple and closely positioned modification sites (Landrieu et al. 2006; Selenko et al. 2008; Theillet et al. 2012b, 2013; Cordier et al. 2012). Success of the method stems from the inherently quantitative nature of NMR spectrum and the possibility to resolve signals simultaneously for both phosphorylated (p) and unmodified (u) states. For serine and threonine residues, the amides signals in the ^1^H–^15^N correlation spectrum shift upon phosphorylation to a distinct region, which is usually empty for non-phosphorylated proteins. This significantly simplifies the resonance assignment and removes signal overlap even in large and disordered systems (Landrieu et al. 2006). Amides of tyrosine residues also change their positions upon phosphorylation. However, shifts of the signals are not so dramatic as for serine and threonine and both p- and u-species often overlap with other protein amide peaks, some of which are also affected by phosphorylation. This was the case in our experiments where we used the Src type tyrosine kinase *Fyn* to phosphorylate the ITAM tyrosines present in the cytosolic domain of CD79b. In Fig. 4a it can be seen that signals of tyrosines Y18 and Y29 strongly overlap with other residues in the overlaid 2D ^1^H–^15^N HSQC spectra of both phosphorylated (pY) and unmodified (uY) forms. Crowding in the 2D spectra complicates monitoring of individual peak intensities during the phosphorylation reaction and thus hinders detection of phosphorylated species and quantitative kinetic analysis for individual amides. In contrast, tyrosine signals and most other residues are well resolved in the 3D HNCO spectrum (Fig. 4b) and can be used for site-specific analysis. Figure 5 shows time evolution of the signal intensities for uY18/pY18, uY29/pY29 and directly adjacent residues during the reaction.

With two phosphorylation sites present in CD79b, we can expect to see up to four forms of the protein during the reaction: uY18–uY29, pY18–uY29, uY18–pY29, and pY18–pY29. Correspondingly, an amide affected by both phosphorylation states may show up to four distinct peaks. In our experiments, however, maximum two peaks were observed for individual residues in the course of the reaction. In all cases, the sum of the two peaks was constant over the reaction time. These observations do not exclude presence of all four phosphorylation species in solution and may correspond to a situation where each amide senses phosphorylation of at most one site. Indeed, an u-, p-state chemical shift difference plot (Fig. S2) clearly shows maxima near Y18 and Y29 and a dip between the two tyrosines. Within the experimental errors, the peak intensities follow first order kinetics described by a single exponent without noticeable lag phase. Amides in the vicinity of Y18 and Y29 show distinctly different phosphorylation rates around 0.25 and 0.19 h^−1^, respectively. These observations are consistent with a simple model where phosphorylation of the two CD79b ITAM tyrosines by *Fyn* kinase occurs independently. However, we cannot completely exclude alternative scenarios of ordered phosphorylation (Selenko et al. 2008; Cordier et al. 2012).

## Conclusions

Time-resolved NMR spectroscopy is an invaluable tool in studying dynamics of complex molecular systems. The proposed methodology addresses two major limitations of the multi-dimensional experiments in time resolved applications: the lengthy sampling and low sensitivity in short experiments. The former is achieved with non-uniform sampling and fast pulsing BEST-TROSY type experiments. The sensitivity is improved with co-MDD analysis exploiting prior knowledge about signal relaxation properties and redundancy of the peak positions and line shapes over the series of the spectra obtained at different time points of the kinetic process. The new method is applicable to almost any type of experiment with dimensionality higher than one. By allowing usage of multidimensional experiments in time-resolved studies the presented approach enables simultaneous monitoring of multiple spectroscopic parameters including chemical shifts of several atoms, relaxation rates, etc.

## Electronic supplementary material

Below is the link to the electronic supplementary material.

Supplementary material 1 (DOCX 963 kb)
