# Supplementary material for: Time-resolved multidimensional NMR with non-uniform sampling
Source: J Biomol NMR. 2014 Jan 17;58(2):129–39. doi: 10.1007/s10858-013-9811-1 (PMC3929766; doi:10.1007/s10858-013-9811-1)
Supplement: Supplementary file 1 — Supplementary material 1 (DOCX 963 kb) [file 10858_2013_9811_MOESM1_ESM.docx]

**Time-Resolved Multidimensional NMR with non-uniform sampling**

Maxim Mayzel, Joakim Rosenlöw, Linnéa Isaksson, Vladislav Y. Orekhov*

The Swedish NMR Centre, University of Gothenburg, Box 465, 40530 Göteborg, Sweden.

***Corresponding author:**

E-mail: Vladislav.Orekhov@nmr.gu.se

Tel. +46 31 786 3886

Fax: +46 31 786 3880

Supplementary Material

**Figure S1.** Amide hydrogen-deuterium exchange for ubiquitin. Experimental points reflect peak intensity measured in individual time windows sliced from the 3D BT-HNCO spectrum and processed with co-MDD. Size of the first window *WS_0_* was 16 points and each next window increased by 5%, i.e. *WS_i+1_* = 1.05 *WS_i_*. Lines and exchange rates correspond to best fit to Equation 3. The residue numbers and rates (in min^-1^) of the calculated HD-exchange are annotated in the panels.

**Figure S2.** CD79b chemical shift perturbation by phosphorylation. Chemical shift difference between unmodified and phosphorylated forms (indexes *u* and *p*, respectively) is calculated as

$\boldsymbol{\Delta\delta=}\sqrt{{\left( {HN}_{u}-{HN}_{p} \right)^{2}}/{\sigma_{HN}^{2}}+{\left( {CO}_{u}-{CO}_{p} \right)^{2}}/{\sigma_{CO}^{2}}+{\left( {NH}_{u}-{NH}_{p} \right)^{2}}/{\sigma_{NH}^{2}}}$,

where $\sigma_{HN}=$ 0.63 ppm, $\sigma_{CO}=$ 1.92 ppm, and $\sigma_{NH}=$ 3.89 ppm are mean over amino acid type chemical shift dispersions calculated from BMRB NMR statistics (http://bmrb.wisc.edu/ref_info/statsel.htm).

**Table S1.** Ubiquitin amide H/D exchange rate constants at 20 ◦ C and pH = 6.0.

| **Residue** | k_ex_ (min^-1^)^a^ | k_ex_ (min^-1^)^b^ | T_1/2_ (min)^c^ |
| --- | --- | --- | --- |
| **07Thr** | 3.5±0.2 10^-2^ | 3.4±0.1 10^-2^ | 20.3 |
| **13Ile** |  | 4.9±1.7 10^-4^ | 1426.2 |
| **16Glu** | 1.4±0.2 10^-1^ | 1.6±0.3 10^-1^ | 5.1 |
| **18Glu** | 1.9±0.1 10^-3^ | 2.0±0.1 10^-3^ | 350.1 |
| **20Ser** | 1.7±0.4 10^-1^ | 1.5±0.5 10^-1^ | 4.0 |
| **22Thr** | 5.5±1.4 10^-4^ | 5.7±1.1 10^-4^ | 1213.9 |
| **25Asn** | 1.6±0.1 10^-3^ | 1.7±0.1 10^-3^ | 415.1 |
| **31Gln** | 8.6±0.3 10^-3^ | 8.8±0.3 10^-3^ | 78.8 |
| **32Asp** | 3.0±0.1 10^-2^ | 3.1±0.2 10^-2^ | 22.7 |
| **33Lys** | 7.0±0.6 10^-2^ | 6.5±0.6 10^-2^ | 10.0 |
| **34Glu** | 1.1±0.0 10^-2^ | 1.2±0.0 10^-2^ | 58.7 |
| **35Gly** | 2.6±0.2 10^-2^ | 2.7±0.1 10^-2^ | 25.3 |
| **36Ile** | 1.8±0.2 10^-3^ | 2.0±0.2 10^-3^ | 350.1 |
| **40Gln** | 3.0±0.2 10^-2^ | 3.1±0.1 10^-2^ | 22.2 |
| **41Gln** | 6.6±0.2 10^-3^ | 6.9±0.2 10^-3^ | 100.2 |
| **42Arg** | 2.6±0.1 10^-3^ | 2.7±0.1 10^-3^ | 258.6 |
| **43Leu** | 1.5±0.1 10^-2^ | 1.6±0.1 10^-2^ | 45.6 |
| **45Phe** | 1.2±0.1 10^-3^ | 1.2±0.1 10^-3^ | 559.0 |
| **48Lys** | 2.2±0.1 10^-2^ | 2.3±0.1 10^-2^ | 30.3 |
| **50Leu** | 5.6±2.0 10^-4^ | 8.8±0.8 10^-4^ | 783.2 |
| **51Glu** | 3.0±0.2 10^-2^ | 3.1±0.2 10^-2^ | 22.4 |
| **54Arg** | 1.3±0.1 10^-3^ | 1.3±0.1 10^-3^ | 541.5 |
| **57Ser** | 1.6±0.0 10^-2^ | 1.5±0.0 10^-2^ | 44.4 |
| **58Asp** | 1.5±0.1 10^-2^ | 1.6±0.1 10^-2^ | 43.6 |
| **60Asn** | 4.1±1.2 10^-1^ |  | 1.7 |
| **61Ile** | 1.6±0.1 10^-3^ | 1.7±0.1 10^-3^ | 398.4 |
| **62Gln** | 6.2±0.3 10^-2^ | 5.9±0.4 10^-2^ | 11.2 |
| **64Glu** | 2.9±0.1 10^-2^ | 2.8±0.1 10^-2^ | 24.1 |
| **65Ser** | 4.5±0.2 10^-2^ | 4.4±0.3 10^-2^ | 15.5 |
| **66Thr** | 1.2±0.2 10^-1^ | 1.5±0.4 10^-1^ | 5.9 |
| **67Leu** | 2.6±0.1 10^-3^ | 2.6±0.1 10^-3^ | 270.8 |
| **68His** | 1.9±0.1 10^-2^ | 2.0±0.1 10^-2^ | 33.8 |
| **69Leu** | 1.0±0.1 10^-3^ | 1.2±0.1 10^-3^ | 563.5 |
| **71Leu** | 1.5±0.2 10^-1^ | 1.9±0.3 10^-1^ | 4.7 |

**^a)^** The first window size *WS_0_* was 16 points and each next window increased by 5%, i.e. *WS_i+1_* = 1.05 *WS_i_*.

**^b)^** 64 points constant time-window

**^c)^** Half time for amide proton exchange, T_1/2_ = ln(2)/k_ex_, where k_ex_ corresponds to the value with lowest fitting error
